# Agent-based modeling of competence phenotype switching in *Bacillus subtilis*

**DOI:** 10.1186/1742-4682-10-23

**Published:** 2013-04-03

**Authors:** Suzy M Stiegelmeyer, Morgan C Giddings

**Affiliations:** 1 Syngenta Biotechnology, Inc., 3054 Cornwallis Rd,, Research Triangle Park, NC 27709, USA; 2 MYS, LLC, 202N 9th St, Suite 303A, Boise, ID 83702, USA; 3 Formerly: College of Arts and Sciences, Boise State University, Boise, ID 83725, USA; 4 Department of Biochemistry & Biophysics, University of North Carolina at Chapel Hill, Chapel Hill, NC 27599, USA

**Keywords:** Agent-based modeling, *Bacillus subtilis*, Bistable switch, Competence

## Abstract

**Background:**

It is a fascinating phenomenon that in genetically identical bacteria populations of *Bacillus subtilis,* a distinct DNA uptake phenotype called the competence phenotype may emerge in 10–20% of the population. Many aspects of the phenomenon are believed to be due to the variable expression of critical genes: a stochastic occurrence termed “noise” which has made the phenomenon difficult to examine directly by lab experimentation.

**Methods:**

To capture and model noise in this system and further understand the emergence of competence both at the intracellular and culture levels in *B. subtilis*, we developed a novel multi-scale, agent-based model. At the intracellular level, our model recreates the regulatory network involved in the competence phenotype. At the culture level, we simulated growth conditions, with our multi-scale model providing feedback between the two levels.

**Results:**

Our model predicted three potential sources of genetic “noise”. First, the random spatial arrangement of molecules may influence the manifestation of the competence phenotype. In addition, the evidence suggests that there may be a type of epigenetic heritability to the emergence of competence, influenced by the molecular concentrations of key competence molecules inherited through cell division. Finally, the emergence of competence during the stationary phase may in part be due to the dilution effect of cell division upon protein concentrations.

**Conclusions:**

The competence phenotype was easily translated into an agent-based model – one with the ability to illuminate complex cell behavior. Models such as the one described in this paper can simulate cell behavior that is otherwise unobservable *in vivo*, highlighting their potential usefulness as research tools.

## Background

The competence state exhibited by *Bacillus subtilis* is an example of a bacterial phenotype driven by changes in gene and protein expression states rather than changes in genotype [1]. Competence is a DNA uptake mechanism that appears to be a cell survival strategy for either procuring new genetic information or obtaining DNA as food. The emergence of competence is correlated with high cell density and nutrient limiting conditions [1]. In those conditions, approximately 10–20% of a *B. subtilis* population will express the competence phenotype [1]. The mechanism by which a small fraction of the population becomes competent is presently attributed to the variable expression of specific genes, i.e., genetic “noise.” “Noise” in gene expression at present is poorly understood.

**Figure 1:**
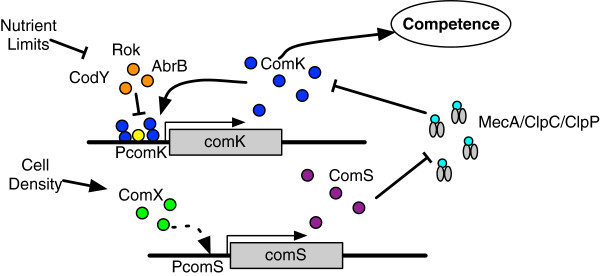
**Competence gene regulatory network.** Significant quantities of ComK will activate downstream competence genes. ComK expression is regulated pre-transcription by repressor proteins and regulated post-translation by degradation by the MecA/ClpC/ClpP protease complex, where ComS competes with ComK to bind to the MecA adapter protein. Increased ComS production will then decrease degradation of the ComK protein. ComK is also upregulated by binding to its own promoter to enhance its further expression through an autoregulatory loop. The transcription regulator DegU is shown in yellow.

The competence phenotype in *B. subtilis* is driven by bistable expression of the *comK* transcription factor [2], which is involved in a feedback loop that regulates its own expression and controls the downstream expression of competence genes, as shown in Figure 1. Bistability refers to a mechanism consisting of two stable genetic regulatory states, in this case ‘ON’ or ‘OFF’ [3-5]. Accumulation of ComK protein (the ON state) enables downstream transcription of the DNA transport genes [6] that lead to the observed competence phenotype. Since *comK* is a “switch” that drives a key phenotypic state, its expression is highly regulated [7] at both the transcript and protein levels (Figure 1). The ability of a bistable switch to transition from one state to another seems to be governed by random variations in biochemical reactions [2,8,9], as we see here with the variable expression of competence regulatory genes.

Stochastic intracellular molecular interactions and environmental inputs like changing nutrients and cell density are known to initiate phenotype switching and are sources of noise [3,10-12]. Yet, the precise molecular interactions driving variable gene expression are difficult to capture in a typical laboratory environment. To educate ourselves about the molecular interactions involved in the competence phenotype, and to speculate if simple temporal and spatial molecular interactions also contribute to genetic noise, we developed a virtual model of a simplified *B. subtilis* cell in a cell colony-like environment.

A variety of mathematical modeling methods have been employed in an attempt to understand the nature of the competence switching process [2,13-15]. These models have addressed the stochastic nature of competence by modeling noise in the system with varying degrees of specificity, using pre-defined noise terms and the Gillespie stochastic modeling algorithm [16]. However, to model noise, a stochastic process, we felt that employing a modeling technique that is itself inherently stochastic may offer additional insights about the nature of competence.

We decided to employ an alternative modeling technique – agent-based modeling (ABM). We hypothesized that the randomly interacting agents of an ABM would be suitable for modeling the genetic noise observed in the competence switch. In our ABM, agents simulate large molecules like proteins, RNAs, and DNAs. Agents interact with each other and their environment based on a set of well-defined rules, either literature-based or biologically intuitive.

Recently, ABMs have been used with great success to model phenomena in bacteriology such as biofilm development [17-21], the transmission dynamics of antibiotic resistance [22], and antibiotic resistance mechanisms in *Staphylococcus aureus*[23]. We recently reported the use of ABMs as applied to the chemotactic switching system in *Escherichia coli*[24].

Here we report the ABM we designed to mimic the *B. subtilis* competence phenotype. The model was tuned to simulate a colony of *B. subtilis* cells where competence emerged in 10–20% of the population. We make the following observations and predictions about genetic “noise”: a) random spatial-temporal interactions lead to competence, b) daughter cells which inherit portions of the competence machinery are more likely to exhibit the competence phenotype, and c) dilution events like cell division inhibit competence emergence until stationary phase. The resulting model’s source code is openly available for exploration at http://www.giddingslab.org/software, for use with the open-source modeling platform Repast Simphony version 1.2 [25].

## Model implementation

### Modeling environment and overview

**Figure 2:**
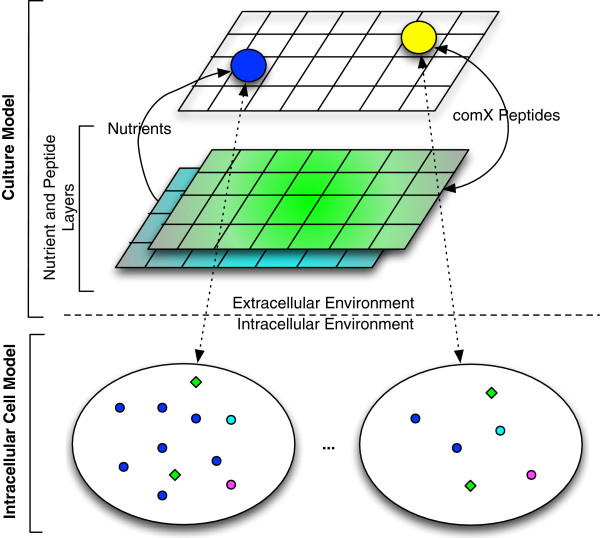
**Multi-scale ABM of competence.** This is a bottom-up ABM. The bottom layer represents the intracellular ABM implementing the competence network, while the upper layer represents the cell culture environment. The two colored layers in the cell culture environment represent the ComX quorum-sensing pheromone and nutrient layers from which cell agents (blue and yellow) consume molecules. The yellow cell agent indicates a cell exhibiting competence.

A multi-scale ABM mimicking the biology of the competence switch was developed using Repast Simphony version 1.2 [25], a Java based, open-source, ABM framework that facilitates model development. Figure 2 gives a conceptual overview of the model. The bottom layer of the model, the intracellular model, is an ABM of the *B. subtilis* cell simulating the intracellular competence regulatory network in a 3-D environment focused on the regulation and production of ComK and ComS proteins (Figure 1). At the top layer of the model, the culture model, the *B. subtilis* cell ABM acts as an agent in this layer simulating bacterial chemotaxis, cell division and death, nutrient consumption, and ComX peptide consumption and production. The nutrient layers depicted in the figure demonstrate the nutrient and ComX peptide simulation in the 2-D grid environment of the culture model. Essentially, we have built an ABM-within-ABM, where the ABM of the bottom layer is an agent in the top layer.

At simulation start, agents were randomly placed in a grid-like environment. Agents can only occupy one cell in the grid at a time and their movement is limited to neighboring adjacent cell locations. Agents interact with the grid environment or other agents by stochastically executing rules (Table 1).

**Table 1:** Agents, the rules they execute, and the agents they interact with

**Agent**	**Rules**	**Interacts with**
Promoter	transcription	ComK, ComX, DegU, Repressor
Repressor	move, bind	Promoter
Ribosome	move, bind, translation	mRNA
mRNA	move, death	Ribosome
ComK	move, bind	Promoter, ComK, MecA
ComS	move	MecA
ComX	move, bind	Promoter
DegU	move, bind	Promoter
MecA	move, bind	ComK, ComS, ClpC/ClpP
ClpC/ClpP	move, bind, death	MecA
Culture Model	diffusion	Nutrients, ComX Peptides
Cell ABM	move, shove, generatePeptide, consumePeptide, consumeNutrients, life, death	Nutrients, ComX Peptides

Time in the model was represented as discrete updates to the state of all agents in the model. A time step was completed when all agent rules had been attempted in a random order. Rules for both the cellular and intracellular models are handled in this manner, keeping both models on the same time scale.

Nutrients and the comX peptide were mathematically modeled using diffusion equations adapted to the ABM environment (Figure 2). The model uses continuous equations due to the high concentrations of nutrients and eventual comX. Concentrations are monitored in each cell of the grid environment and diffusion to adjacent cells is calculated by the equations described below.

### Rule execution

In the model, agent movement and interactions with other agents are defined by rules. Rule execution is stochastic and subject to meeting a probability threshold after a random draw from the uniform distribution (see Parameter Estimation).

For example, if two molecules have been shown to bind biologically with a high affinity, then their interaction probability in the model will be high. Hamoen et al. [26] reported that ComK may bind to another ComK to form a homodimer. This is represented in the model using an interaction probability ρ (Table 2), with the following rule executed whenever a ComK finds itself next to another ComK agent, listed as *neighbor* here:

**if***neighbor* = ComK **then**

random = generate random number between 0 and 1.

**if** random < ρ **then**

neighbor now moves with ComK

end if

end if

**Table 2:** Initial agent quantities

**Agent**	**Starting number at t0**
Promoter	12
Repressor	2
Ribosome	80
mRNA	0
ComK	0–12
ComS	0–12
ComX	0–12
DegU	0–12
MecA	20
ClpC/ClpP	20
Cell ABM	20

If the probability threshold is not met, then the rule is not executed. The process is repeated at each time step, until eventually the probability threshold is met and the rule is executed.

### Parameter estimation

The parameter space is quite large for this model and is divided into three types of parameter estimates: grid environment size, initial number of agents, and rule interaction probabilities. The parameters were estimated using a random sweep parameter estimation technique, where: (1) initial parameter estimates were made based on biological insight (see next paragraph), (2) repeated simulations were then run where parameter values were randomly changed, and (3) final parameter values were determined from simulations which best mimicked experimentally obtained results, as reported by Maamar et al. [2].

Rule probabilities were initially estimated based on known interactions and then fit as simulations were run. For instance, if two molecules had a high affinity, then a high probability of interaction (e.g. 0.8) was estimated. For two molecules with a low affinity, a low probability (e.g. 0.2) was estimated. As simulations were run, rule probabilities were increased or decreased to speed up or slow down, respectively, a particular molecular reaction.

### Intracellular agent-based model

The intracellular model was designed to closely mimic the biology of the competence regulatory network and is described as follows in terms of the biology. As stated previously, an abundance of ComK triggers the switch of a *B. subtilis* cell to exhibit the competence phenotype. As such, the model was designed around the regulation of *comK* at transcription and post-translation. Agents were translated from the biological model to represent ComK, ComS, DegU and MecA proteins, the ComX peptide, ribosomes, *comK* and *comS* transcripts, repressors, *comK* and *comS* promoter sites, and the ClpC/ClpP protease, as shown in Table 1. At startup of the model, a random number of ComK, ComS, ComX, and DegU agents was generated from the value ranges given in Table 2 for these agents. Any exceptions to this are described in Results. The agents interact with one another and their environment in a 3-D grid of 40 × 40 × 40 cells. Each agent’s behavior is defined by a set of rules, summarized in Tables 1 and 3 and described further below.

**Table 3:** Interacting agents, rule execution probabilities, and behavior

**Rules**	**Interacting agents**	**Probability**	**Resulting action**
bind	Repressor-Promoter	0.5	Repressor + Promoter
	DegU-Promoter	0.5	DegU + Promoter
	ComX-Promoter	0.5	ComX + Promoter
	ComK-Promoter	0.5	ComK + Promoter
	ComK-Promoter-DegU	0.8	ComK + Promoter + DegU
	ComK-ComK	0.8	ComK + ComK
	Ribosome-mRNA	0.9	Ribosome + mRNA
	ClpC/ClpP + MecA-ComK	0.6	ClpC/ClpP + MecA + ComK
	ClpC/ClpP + MecA-ComS	0.7	ClpC/ClpP + MecA + ComS
	ClpP/ClpC-MecA	0.5	ClpC/ClpP + MecA
consumePeptide	Cell	0.8	New ComX
death	mRNA	0.0001	Remove mRNA
	ClpC/ClpP + MecA + ComK/ComS	0.5	Remove ComK or Remove ComS
	Cell	0.0001	Remove Cell
dissociation	Repressor	0.0001	Repressor-Promoter
	DegU	0.0001	DegU-Promoter
generatePeptide	Cell	0.8	
Life	Cell	0.8	New Cell if not starving
	Cell	0.0001	Remove Repressor if starving
Move	Cell	0.5	chemotaxis
Move	ComK, ComS, ComX, DegU, MecA, ClpC/ClpP, mRNA, Ribosome, Repressor	Random walk, see text	
Shove	Cell	-	
transcription	Promoter	0.0001	New mRNA
	Promoter + ComK dimer	0.001	New mRNA
	Promoter + ComK tertramer	0.5	New mRNA
	Promoter + ComX	0.5	New mRNA
translation	Ribosome + mRNA	0.5	New ComK or New ComS

Agent-agent represents unbound neighboring agents, while agent + agent represents bound agents moving together.

### *ComK* transcription and translation

ComK binds at its own promoter as a tetramer, acting as its own transcription factor [6,26]. Random transcription of *comK* is a key factor in the build-up of large amounts of ComK, which triggers transcription of the DNA transport (competence) genes [2,27]. DegU binds to the *comK* promoter and strongly stimulates binding of ComK dimers to the *comK* promoter [28,29]. More specifically, DegU binds in between the two ComK dimer binding sites and may possibly facilitate tetramerization of ComK at the *comK* promoter site by partial unwinding and bending of the DNA helix [29]. Transcription can also occur in the absence of ComK [30]. It is estimated that a cell exhibiting competence has on average 50,000 ComK dimers during stationary phase [2].

To simulate *comK* transcription in the intracellular model, agents representing ComK, the *comK* promoter, and DegU are modeled. Binding of ComK agents to the *comK* promoter agent is contingent upon what agents, if any, are already bound; in the model, this is reflected in the creation of binding rule probabilities (Table 3) calculated during the parameter estimation process. If no agents are bound to the promoter agent at the time a ComK dimer encounters it, the lowest binding probability is used. If DegU is already bound to the promoter agent, a higher probability of binding is used. Finally, the highest probability is used for binding the second ComK dimer if DegU and another ComK dimer are present.

Transcription probabilities follow in a similar fashion (Table 3). Transcription occurs at a very low probability when no ComK is bound – its probability increases with the addition of one or two bound ComK molecules. Activators and repressors (described in *comK* Transcriptional Regulation) disassociate upon successful completion of transcription. After the transcription rule is executed, an mRNA agent is generated, which will persist until it randomly degrades as defined by its “death” rule. Since the *comK* transcript has a strong Shine-Delgarno ribosome initiation sequence, the binding and translation probability of the ribosome agent is very high if it encounters a *comK* transcript (Table 3). Therefore, the presence of a single transcript is often enough to lead to the production of several ComK proteins, as reported in Maamar et al. [2].

### *ComK* transcriptional regulation

At present, there are three known *comK* transcriptional repressors: Rok, AbrB and CodY. The *comK* promoter site allows simultaneous binding of AbrB and ComK [31]. The presence of AbrB acts to prevent binding of RNA polymerase, as does CodY [32]. In the model, all three repressors are represented by a generic repressor agent that binds to the *comK* promoter agent. Nutrient limiting conditions down-regulate both AbrB and CodY, thus repressor agents are removed from the model when the cell agent reaches a starvation condition described below. Successful transcription and translation events eventually lead to the build-up of ComK, triggering the feedback loop shown in Figure 1[2,27].

### *ComS* transcription and regulation

ComS is a protein produced in response to quorum sensing (cell density) [33,34]. Transcription of *comS* occurs in response to the quorum-sensing signaling pathway initiated by the ComX peptide. ComX is produced by the cell at a constant rate during growth and accumulates in the cell medium, reflecting cell density [1,35]. For the purposes of the cell model, ComX is the post-translationally modified and cleaved extracellular end product absorbed by the cell. ComX initiates the activation of several proteins, which in turn initiate transcription of *comS*[1]. The model represents this process by utilizing only a ComX agent. When the ComX agent binds to the *comS* promoter, *comS* is activated. The interaction probability given in Table 3 controls this rule, which represents a simplified version of the actual activation pathway. In reality, ComX is not the actual *comS* transcription factor. In addition, regulation of the quorum-sensing pathway is modeled by assuming a repressor agent acts at the *comS* promoter site, as shown in Table 2.

### ComK and ComS post-translational regulation

The MecA/ClpC/ClpP protease complex degrades both ComK and ComS proteins. There are approximately 300 MecA/ClpC/ClpP molecules in a cell [2]. MecA, an adapter protein, binds with either ComK or ComS, targeting the proteins for degradation by ClpC/ClpP [36]. ComS competes with ComK for binding with MecA, with ComS having a higher affinity than ComK [37]. If ComK is bound to MecA upon encountering ComS, ComK disassociates, targeting ComS for degradation instead. Because ComK is positively auto-regulated, protection from degradation by ComS results in an explosive increase in ComK synthesis [36,37]. In this fashion, the up-regulation of ComS due to quorum sensing leads to an increased accumulation of ComK, and the cell transitions to the competence state. Thus, in the model, when ClpP/ClpC + MecA + ComK-bound agents encounters a ComS agent, ComK is released and ComS is bound.

This system was represented by implementing the interaction probabilities shown in Table 3. Since ComS has a stronger affinity to the adapter protein MecA, a higher binding probability is used for association with ComK during execution of the binding rule.

### Intracellular model rules

#### Move rule

The move rule simulates a random walk of an agent through the 3-D landscape to imitate molecular diffusion and Brownian motion. Each successful execution of the rule results in a one-step move of an agent to a randomly selected neighboring cell on the grid. In the 3-D grid of the intracellular model, there are 26 possible adjacent neighboring cells into which an agent could attempt to move. If the randomly selected cell is occupied, then the agent remains in place.

#### Bind rule

This rule is used for molecules that bind with other molecules when encountering another in a neighboring cell during the course of the random walk. For each agent, the bind rule searches for other agents at adjacent grid positions. When there is an adjacent agent for which a binding rule is defined, the two bind by moving together if the rule probability is met. An exception occurs when a MecA agent already bound to ComK encounters ComS – in this instance, ComK dissociates in favor of ComS. Agents that bind with one another are provided in Table 3 along with their associated rule probabilities determined during the parameter estimation process.

#### Transcription rule

The transcription rule is executed by the promoter agent and results in the production of mRNA agents. The success of this rule depends on what is already bound to the promoter agent, to match the biology described above and as specified in Table 3.

#### Translation rule

The ribosome agent executes the translation rule to generate ComK agents or ComS agents depending on the type of the mRNA agent to which it is bound (Table 3).

#### Death rule

Both the mRNA and ClpP/ClpC agents implement a death rule. For the mRNA agent, the death rule represents the random degradation of mRNA that occurs in the cell and is executed when unbound. In the case of the ClpP/ClpC protease complex, the death rule initiates the removal/death of the bound ComK or ComS agent. Table 3 lists the rule probabilities.

### Culture agent-based model

In the cell culture model, agents interact with one another and their environment in a 2-D grid of 40 × 40 cells. Rules are executed in a random order for each iteration of the model, and probabilities calculated during the parameter estimation process determine whether or not a rule executed, as shown in Table 3.

#### Cell culture agents

There is technically only one agent type in the cell culture model, a cell ABM whose internal workings are described above. In the culture model, the cell ABM acts as an agent with the rules specified in Table 1. It interacts with its environment by consuming nutrients, and with other cell agents through production and consumption of the ComX pheromone. Nutrients and the ComX peptide are modeled by diffusion equations due to their high concentrations. Due to the way Repast Simphony is structured, the culture plate itself acts as a layer of immobile agents to allow for the execution of the diffusion rule. Consumption of either nutrients or ComX peptides by the cell agent are fed to the internal cell models, leading to the reduction of repressor agents or the increase of ComX agents, respectively. When more than 20 ComK agents were generated, the cell was considered to have reached competence (shown as a change in color in Figure 2). This threshold is an estimated parameter and was set to this small value to limit the consumption of computational resources.

#### Cell growth equation

The cell agent implements a growth function to control cell growth, division, and death. The growth function is based on the Logistic Map function: *m*_*n+1*_ *= μm*_*n*_*-μm*^*2*^_*n*_*/k*, where *μ = 0.0058* is the growth rate [38], *m* represents the energy of the cell, and *k* is the maximum energy [39]. *m*_*n*_ is the value of the energy function at iteration *n*. In the culture ABM, *m*_*0*_ was initialized to 5 and *k* was set to 16. The *μm*_*n*_ term of the equation signifies an energy gain that occurs when consuming nutrients. The *μm*^*2*^_*n*_*/k* term signifies a decrease in energy, as it is assumed that basic metabolism within the cell consumes energy. The calculation of the growth function is split across two rules that are described further below: move and consumeNutrients. The life rule uses the value of the equation to determine whether the cell had accumulated enough energy to divide or not. In the move rule, energy is reduced by *μm*^*2*^_*n*_*/k*. If there are no nutrients at the agent’s location, there is no energy increase.

Once nutrients reach a low level (<1) such that cell agents could no longer consume them, the energy level steadily decreases instead of increasing via the move rule. At this stage, the cell growth equation is altered and energy is reduced by *d/(k/2)*, where *d* is the death rate, *d = 0.002*.

### Cell culture rules

#### Diffusion rule

The cell culture executes essentially one global rule, the diffusion rule. This rule executes the Repast Simphony diffusion algorithm on both the nutrient and peptide value layers, based on Rucker’s diffusion equation for Cellular Automata [40]. For each cell in the grid, the difference between the current cell value and the weighted average of neighboring cells is calculated. The resulting value is then multiplied by the diffusion constant and added to the current cell value, thus ensuring that the concentrations within the grid cells move down the concentration gradient. The diffusion constant determined during the parameter estimation process is 0.1.

#### Move rule

The cell agent executes a move rule that simulates bacterial chemotaxis so the cell agent moves towards the most favorable nutrient conditions. If nutrients are available, the cell agent remains at its location. If not, the cell agent randomly selects a free neighboring location with a higher concentration of nutrients. The energy reduction portion of the cell growth equation is implemented when this rule is executed due to the assumption that there is a metabolic cost to a cell’s energy at each iteration.

The move rule also determines when nutrients can no longer be consumed at the cell agent’s current location, and thus disables repressor agents within the cell ABM. A randomly selected repressor agent is removed from the model if a probability threshold (0.001) determined during the parameter estimation process is met.

#### Consumption rules

The consumePeptide and consumeNutrients rules cause the consumption of one molecule from the grid location of the cell agent. When a ComX peptide is consumed, it is added as an agent to the cell ABM. When a nutrient is consumed, a gain in energy occurs, as described above. The consumeNutrient rule is executed at every iteration, while the consumePeptide rule is executed every 50 iterations, if a probability of 0.8 is met.

#### GeneratePeptide rule

The generatePeptide rule produces one molecule of the ComX peptide, which is added to the concentration at the current grid location. This rule is an approximation of the constant production of the ComX peptide described in the literature [1]. Unlike the other rules, the generatePeptide rule is executed every 100 iterations and a ComX peptide is generated with a probability of 0.8. This number of iterations was selected during the parameter estimation process to ensure a gradual production of the peptide. The ComX peptides produced in this manner are independent of the ComX agents residing within the cell ABM agent.

#### Life rule

The life rule determines whether the cell is ready to divide when the energy exceeds a predefined threshold of 15, which is one fewer than *k,* the maximum energy (see growth equation parameters above). Cells that exhibit the competence state do not divide. During the division process, a new cell ABM is created and added to the grid in a neighboring, adjacent grid location. The daughter cell receives half the energy of the parent cell, thereby reducing the parent’s energy by half. The daughter cell will follow intracellular model startup and agent initialization as described in previous sections and in Table 2. However, some of the initial agent quantities are handled differently.

To model inheritance, an arbitrary plane is randomly chosen which bisects the parent cell ABM through its center. ComK, ComS, ComX and mRNA agents ‘above’ the plane remain in the parent cell and agents ‘below’ the plane become a part of the daughter cell. The daughter cell is then placed in a randomly selected grid location adjacent to the parent cell. If that location is occupied, then the shove rule is executed. The simulation assures that the associated genes (promoter agents) are equally distributed between mother and daughter to represent the non-stochastic genome segregation in real cells.

The life rule is also responsible for determining the death of a cell agent. The effect of this rule is shown during the death phase of the bacterial growth curve. If the cell agent’s energy becomes very low (< 0.5) and the probability threshold of 0.0001 is met, then the cell agent ‘dies’ and is removed from the model.

#### Shove rule

The shove rule is intended to displace cell agents one step to an adjacent, randomly selected, neighboring position if more than one cell agent occupies its current location. As each agent executes this rule, a one step displacement of cell agents ripples through a group of adjacent cell agents until there is room for all cell agents in the culture model grid.

#### Death rule

**Figure 3:**
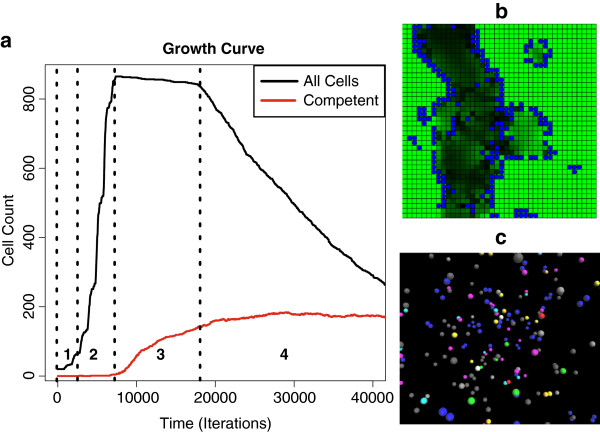
**Bacteria growth curve generated by the model. a**) Bacteria growth curve generated by the model with distinct phases labeled corresponding with known bacterial growth phases: 1-lag phase, 2-exponential growth phase, 3-stationary phase, and 4-death phase. **b**) This is a view of the cell culture model where nutrient counts are shown in green and cells in blue. Dark patches show regions where nutrients have become depleted due to consumption by cellular agents. **c**) Agents are shown interacting inside the intracellular model.

This rule models the random die-off of cells with a high metabolism when nutrients become insufficient. This rule is responsible for the slight dip observed in the growth curve during stationary phase, Figure 3a. The death rule executed every 50 iterations instead of every iteration to limit the death rate. The cell agent ‘dies’ when a probability threshold of 0.0001 is met and when the cell’s energy is within 0.5–7.5 (half the energy threshold needed for division).

## Results

### Intracellular model

**Figure 4:**
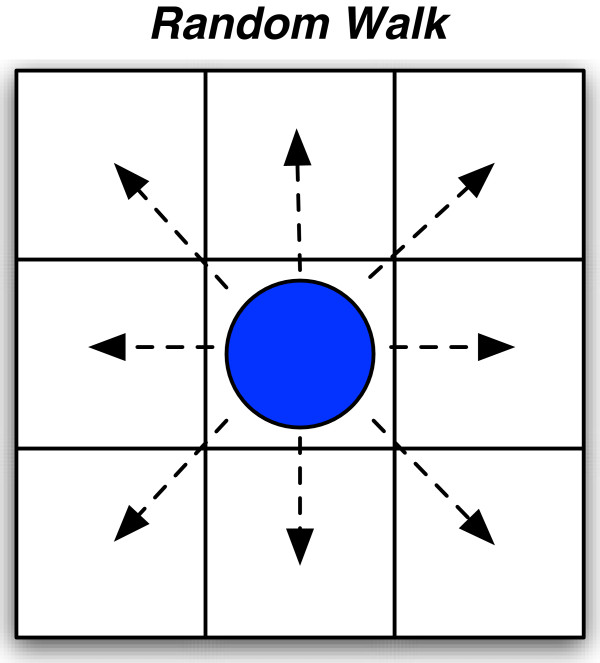
**Agent movement follows random walk.** Movement of agents based on Brownian motion and simulated by a random walk. An agent will randomly select an adjacent cell and move to that new location.

To predict possible mechanisms of the noise that drives competence expression, we developed a 3-D intracellular ABM to represent the key molecular players in competence switching in *B. subtilis*, and to mimic their interactions (Figure 1). In the ABM, we explicitly represented proteins, genes, transcripts, and ribosomes as agents that could move and interact in a simulated cellular environment. To realistically model the bottom-up behavior of the system, the model included the transcription and translation of genes to proteins. Agent movement was based on Brownian motion simulated by a random walk (Figure 4). When agents encountered other agents in a simulation, they interacted stochastically according to behaviors defined by rules discussed in the Model Implementation section (Tables 1, 2, 3). As it is known that proteins can localize to specific areas within a bacteria cell [41], the bacteria cell model represents the portion of the cell where the DNA localizes. We assumed for the purposes of this model that the proteins of interest diffused randomly in this region until captured by a protease or bound as a transcription factor. We also assume that the volume of this region remains constant. The experiment began with an initial run of 20 intracellular models to reduce the consumption of computational resources.

At model start-up, agents were placed at random locations within the simulated 3-D grid environment, Figure 3c. Several simulations were run to assess outcomes given different initial configurations.

### Cell culture level modeling

One goal of the study was to leverage the intracellular model into a multi-scale model of cell culture level interactions, to examine the effects of signaling and competition for nutrients on the outcome of competence. The cell-level model consisted of whole cell agents representing a cell’s interaction with external environmental factors such as nutrients and the quorum-sensing pheromone ComX. Agents were placed randomly in a 2-D grid environment (Figure 3b), much like the random placement of agents in the 3-D intracellular tier (Figure 3c).

In this multi-scale model, external environmental conditions influenced the intracellular conditions and cell-level outcomes, and intracellular conditions fed back upon the environment and other agents within it. For all multi-scale simulations, the initial intracellular model quantities of ComK, ComS, and ComX agents were randomly determined from thresholds as explained in Model Implementation. There were no *comK* mRNA agents placed at the start of a model run, but these agents were randomly generated via transcription during a simulation.

### Competence

Successful execution of transcription and translation rules eventually led to the build-up of ComK, triggering the feedback loop shown in Figure 1[2,27]. As competition between ComK and ComS proteins is vital in determining the competence state, their production was monitored. Considerably fewer molecules were used in the simulations compared to actual biology [42] due to limitations in computational resources. As a result, a cell was considered to be in the competence state when it had more than 20 ComK proteins – a value determined during the parameter search stage that met the 10–20% competent cells criterion. In reality, approximately 50,000 ComK dimers have been observed in a competent cell [2].

### Competence outcome at the intracellular level: the impact of random spatial-temporal agent arrangement

Initial simulations of the intracellular model were run repeatedly outside of the full model environment to test if spatial arrangement of molecules might contribute to the resulting competence state. Intracellular model simulations were run with identical parameters and concentrations of agents (Table 2, maximum values for ComK, ComS, ComX and DegU were used in these simulations), the sole difference between each run being the random spatial placement of the agents before the simulation started. We ran the simulation 100 times to obtain statistics regarding the overall rate of competence emergence.

**Figure 5:**
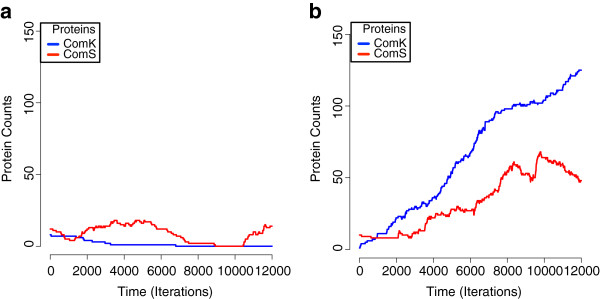
**Identical initial parameters result in two distinct phenotypes.** This figure shows two simulations of the intracellular model with the only difference between them being the random spatial placement of agents in the 3-D grid. **a**) ComS production exceeded ComK and competence did not occur. **b**) In a small percentage of simulations (see text), ComK production exceeded ComS and drove its own positive feedback loop resulting in competence.

Figure 5a and b show the ComK and ComS population sizes through time in two separate runs of intracellular simulations. Figure 5a shows a system that remained non-competent and Figure 5b shows a system that reached the competence state. Notably, the two simulations resulted in different outcomes for both the ComK and ComS proteins.

We repeatedly observed that simulations with identical initial parameters and population sizes resulted in significantly different outcomes, suggesting that the initial arrangement of molecules indeed has a strong impact on competence expression. Given the parameter settings, the over-production of ComK was observed in approximately 3–5 out of 100 simulations (Figure 5b) and the production of ComK outpaced repressor protein activity [43]. Though 3–5% is a lower rate of competence expression than the 10–20% that is typically seen in the bacteria, the model was run without explicit simulation of cell growth, division, or starvation conditions that influence a higher density of competence emergence.

Another outcome of the simulations was that the chance encounter of a single *comK* mRNA transcript with a ribosome before it was degraded would produce a ComK protein. This agrees with prior *in vivo* results indicating that an increased probability of competence switching can be driven by fluctuation of the ComK protein by a few ComK mRNA molecules [2]. On average, one mRNA was observed at any given time in the model comparied to > 20 during growth phase and on average 1 mRNA during stationary phase *in vivo*[2]. So cell fate in the ABM was determined simply by the location of the molecule and its chance encounter with a ribosome. These results provide evidence that a random, spatial arrangement of molecules may be a major contributor to the variable *comK* gene expression (noise) underlying the competence phenotype, and could lead to experiments that further decode the enigma of competence expression.

### Competence outcome at the cell culture level: the impact of nutrient limitation

It has been previously shown that nutrient limitation and cell culture density increase the propensity of *B. subtilis* cells to enter the competence state [1]. In our intracellular model, only a small fraction of the simulations ended in the competence state since the model did not include limitations comparable to real-life conditions. However, within actual cell cultures, once stationary phase is reached and nutrients become limited, a much higher fraction of cells emerge with the competence phenotype - as many as 20%. We sought to determine what bottom-up assumptions drove this number by running simulations of the complete model – intracellular ABMs acting as agents in the culture model.

At each simulation run, the culture model was seeded with 20 intracellular ABMs placed in random locations in the 2-D grid environment. Each of the 20 initial intracellular models began with quantities of ComK, ComS, ComX, and DegU determined by a random draw from a uniform distribution from within a defined threshold, and with 0 mRNA agents. Cells grew and divided, with division resulting in a random partitioning of molecular contents among the progeny. All cells were tracked throughout the simulation, and progeny progressed from 20 initial cells to a maximum of 877 cells in one simulation, with 137 (15.6%) showing competence at simulation termination.

Since individual simulations would often result in distinct outcomes, we ran the model repeatedly to obtain average statistics for competence-switching behavior. In six simulations, the initial seed of 20 randomly placed cells produced an average of 867 cells. We limited the available “plate size” and nutrient concentration for culture growth to limit the computing to feasible time spans. For each of the cells produced, a complete intracellular model was running, which meant that a full simulation running on a fast desktop computer took approximately two months or more to complete. Improved parallelism will reduce run times in the future.

Figure 3a shows the growth curve for an example multi-scale simulation, with the resulting count of competent cells as they switched to the competence phenotype. As with all of the simulations, it resulted in a classic bacterial growth curve with the standard phases of bacterial culture: lag phase, exponential growth phase, stationary phase, and death phase (see Additional file 1 for a movie of lag through stationary phase). The simulations also demonstrated an emergence of competence comparable to actual bacterial cultures: an average of 16.3% competent cells emerged in our simulations by the end of stationary phase, in line with stationary growth phase *in vitro* rates of 10–20% [1]. After cell division ceased due to nutrient limitation, *comK* transcripts and protein quantities increased the likelihood of competence transition. Execution halted within the death phase of the growth curve after approximately 43,000 iterations.

### Competence outcome at the cell culture level: epigenetic heritability

Nutrient limitation and cell density does not, however, explain why those particular 10–20% of cells that express competence do so. Following from the result showing that initial spatial distribution of molecules may affect the outcome of the intracellular model, we postulated that some level of epigenetic heritability might exist in competence switching. The rationale is that if a parent cell has an increased quantity of ComK compared to the average, it might be expected that as the cell divides, the resulting progeny may also have elevated ComK levels. Evidence for this type of heritability in the biological system was presented by Veening et al. [44]. The precursor to competence – accumulation of ComK – may lead to an earlier switch than would occur by random chance alone, a result which would be clearly identifiable in our cell culture model.

**Figure 6:**
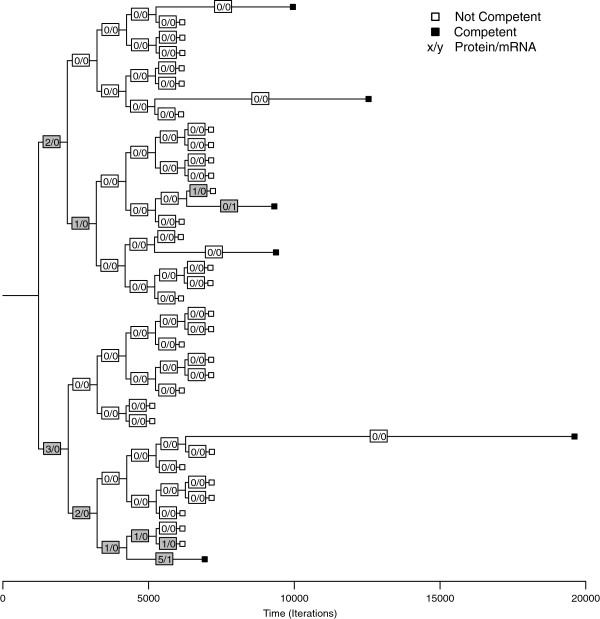
**Inheritance during cell division.** The numbers within the squares indicate actual quantities of *comK* transcripts and ComK proteins. Filled squares indicate the daughter cells that eventually exhibited the competence phenotype, and branch lengths reflect the time (in iterations) until competence was exhibited. Branch lengths of non-competent cells (unfilled squares) were artificially lengthened to make room for the labels; otherwise they would be of zero length. Cells that inherited quantities of ComK proteins and transcripts, combined with intracellular stochasticity, exhibited competence prior to cells that did not acquire ComK. The tree is drawn using R [45] from data captured from the simulation after formatting in Newick tree format.

Each of the initial models was started with quantities of ComK*,* ComS*,* ComX, and DegU determined by a random draw from a uniform distribution within a defined threshold (Table 2). Cells would grow and divide, with division resulting in a random partitioning of molecular contents among the progeny. Figure 6 shows the lineage following a single cell, with ComK protein and transcript levels denoted at each division. Two daughter cells that initially inherited non-zero ComK levels exhibited the competence phenotype significantly earlier than cells that inherited minimal or no ComK. Of the 37 progeny in the lineage shown, six (16%) eventually switched to the competent state, with the first two transitions occurring along the lineages that had elevated levels. In the simulation overall, a cell exhibited competence on average after 4074 ± 2258 iterations after dividing if it had inherited nonzero ComK. Otherwise, a cell exhibited competence on average after 7780 ± 3870 iterations without inheriting. The difference was statistically significant (p < 0.0001), leading us to believe that inheritance contributes to the emergence of competence. This result was consistent from simulation to simulation.

## Discussion

Our interest in phenotype switching derives from studies of antimicrobial-tolerant bacterial phenotypes [46]. These are non-inherited phenotypes that confer tolerance towards many known antibiotic drugs. In bacterial populations, cells exhibiting tolerant phenotypes exist as a small fraction of the population, with the quantity determined in part by growth conditions. For example, stationary phase growth induces an increase in the fraction of phenotypically drug-tolerant cells [47]. However, at the present time, little is known about the mechanisms underlying antimicrobial tolerance in bacteria. So, to explore mechanisms that could explain variable gene expression and to further our knowledge of phenotypic bistable switching, we turned to competence switching in *B. subtilis*, where the key molecular players are known and well-studied.

Our *B. subtilis* ABM is a reduced scale model tuned to reflect the bacteria biology and the competence phenotype. Through the Cell Growth Equation (see Model Implementation section) the model closely approximates bacterial growth curves. In addition, cells only turned competent in bulk once stationary phase was entered. Although quantities of molecular agents modeled were a small fraction of the molecules in a live system, the model was able to approximate a real system. We have found similar results in a separate effort modeling chemotaxis in *E. coli* with an ABM, where preserving biological ratios of molecules was more important than preserving absolute quantities to produce biologically realistic results [24]. However, in the case of this model we found that chance, random interactions between agents had the most effect on the behavior of the model.

In our model, spatial-temporal interactions – not molecular quantities or agent rule execution probabilities – may be the key contributor to the emergence of the competence phenotype, as transcription and translation occur due to chance encounters by the molecules involved. Though it is not yet possible to verify these simulations *in vivo*, the biological system likely shares similar properties where the apparent randomness of the competence transitions is derived directly from the randomness of spatial distribution in competence-related molecules. Noise in a biological system is typically thought of as a series of events, but our results show it is possible that random spatial arrangement of competence-determining molecules may be its major contributing factor.

In addition to random spatial arrangements, random encounters between key agents also appeared to be important in the emergence of competence. It is interesting to note that changing the quantities of the initial number of agents in the model had more of an effect on the model outcome than the rule probabilities themselves. If there are greater numbers of an agent, then there is a higher chance for encounters between agents to occur. During the parameter estimation process, it was observed that changes in the probability values did not greatly impact model outcomes, although higher probabilities did speed up certain reactions (e.g. translation), which resulted in the production of more agents. This seems logical considering that the rule probabilities do not come into effect until two agents encounter one another, which can occur more frequently as the quantities of agents increase. If there are a large number of agents, there are more chances for them to encounter one another. As parameters have been reported to lack sensitivity in many biological models [48], our parameterization method is somewhat un-formalized as we focused more on looking for biological interpretations of our model after it had reached a life-like representation of the competence switch.

The cell division trees that monitored quantities of *ComK* displayed an obvious pattern of inheritance. Cells that inherited higher amounts of *ComK* transcripts and proteins from the parent cell tended to, in turn, pass on higher quantities to their children cells. However, the model also suggests what may be a basic but important facet of the emergence of competence: that dilution of competence-determining molecules during cell division may act to regulate the emergence of competence. Except for the lineages that switched to the competent state early due to inheriting elevated levels of the *ComK* protein, the remaining competent cells only became so after nutrient limitation and repressor agents inhibited cell division long enough for sufficient quantities of *ComK* agents to accumulate. The quantities of molecules inherited in this pathway acted in an epigenetic fashion upon subsequent phenotypic outcomes. This explains why competence in *B. subtilis* typically emerges during stationary phase when nutrients are limited, due to build-up of elements like *ComK* in the competence gene regulatory network.

The model may therefore reveal an important feature of how the growth stage regulates competence by diluting the mRNA and proteins essential to switching to the competent state. Although we have not verified this experimentally, Roostalu et al. [49] reported fluctuations in a reporter protein after cell division that supports the theory that cell division acts as a dilution event for the competence phenotype. During exponential growth phase, not enough time would pass between cell divisions to allow *ComK* to build up to sufficient levels, so competence would not occur. While we cannot know whether this is the complete explanation for how competence is limited to stationary phase in *B. subtilis* cultures, it appears to be a sufficient explanation to guide further experiments.

## Conclusions

This study suffers from the same limitations as any model-based research, in that all models are only *representations* of a biological system. Nevertheless, the design of the model facilitates a thought experiment to represent and clarify the workings behind very complex cell behavior. This discrete model of the competence phenotype provides a readily comprehensible view into each cell’s behavior and provides the ability to monitor the variation of molecular concentrations involved in regulating competence. The resulting model is biologically intuitive, with ready translation from biological facts or hypotheses into the model and back. As we used this model to educate ourselves on the mechanisms of the competence phenotype, we see great potential in ABMs as educational tools in the future because they are straightforward to build, visualize, and comprehend.

## Competing interests

The authors declare that they have no competing interests.

## Authors’ contributions

SMS carried out the design and testing of the model, interpreted the results, and drafted the manuscript. MCG participated in study design and coordination and helped draft the manuscript. Both authors read and approved the final manuscript.

## Supplementary Material

Additional file 1 **Movie of Cell Culture Model.** This movie shows the cell culture ABM initially seeded with 20 cells (blue). Green indicates nutrient-rich cells. Cells grow and divide as they consume nutrients. The formerly nutrient-rich cells darken as nutrient concentrations decrease. Cells move following the nutrient gradient as nutrients are depleted. When cells die, nutrients are released into the environment. The movie halts during stationary phase. Click here for file
